# The Effects of Foods on Blood Lipids in Non-alcoholic Fatty Liver Disease (NAFLD)—A Systematic Review and Meta-Analysis

**DOI:** 10.3389/fnut.2020.613221

**Published:** 2020-12-16

**Authors:** Fredrik Rosqvist, Andreas Rydell, David Iggman

**Affiliations:** ^1^Clinical Nutrition and Metabolism, Department of Public Health and Caring Sciences, Uppsala University, Uppsala, Sweden; ^2^Norslund-Svärdsjö Academic Health Care Center, Center for Clinical Research Dalarna, Falun, Sweden; ^3^Division of Family Medicine and Primary Care, Department of Neurobiology, Care Sciences and Society (NVS), Karolinska Institute, Huddinge, Sweden

**Keywords:** NAFLD, steatosis, cholesterol, triglycerides, diet, food

## Abstract

**Background:** Non-alcoholic fatty liver disease (NAFLD) is associated with dyslipidemia and increased cardiovascular disease risk. Dietary choices may produce profound effects on blood lipids. Thus, the purpose of this study was to investigate which foods modify blood lipids in NAFLD.

**Methods:** Systematic review of published systematic reviews and randomized controlled trials (RCTs). Searches were performed in PubMed, Cochrane Database of Systematic Reviews, and Cochrane Central Register of Controlled Trials, from inception through March 2020. Studies in populations with NAFLD, which provided data on foods or dietary patterns and blood lipids were included, but not weight loss diets, supplements, nor individual nutrients. The strength of evidence was evaluated using The Grading of Recommendations Assessment, Development, and Evaluation (GRADE).

**Results:** No relevant systematic reviews were identified. Eleven RCTs were included in the qualitative synthesis. Two RCTs were included in meta-analyses, regarding the comparison between Mediterranean and Low-fat diets, in which there were no clear effects on either high-density lipoprotein cholesterol or triglycerides, with Low evidence. From single RCTs, there was Moderate evidence for reduced triglycerides by a healthy dietary pattern, compared with usual care; and for reduced total cholesterol by a probiotic yogurt, enriched with *Lactobacillus acidophilus* La5 and *Bifidobacterium lactis* Bb12, compared with conventional yogurt. For all other comparisons, the evidence was considered as Low or Very low.

**Conclusion:** Few studies were identified which reported effects of foods on blood lipids in subjects with NAFLD. The possible beneficial effect of probiotics warrants further study. PROSPERO identifier: CRD42020178927.

## Introduction

Non-alcoholic fatty liver disease (NAFLD) is a prevalent and emerging public health concern, closely associated with obesity, metabolic syndrome, insulin resistance, and type 2 diabetes (1). Individuals with NAFLD frequently present with dyslipidemia, typically elevated fasting plasma triglycerides and/or low high-density lipoprotein (HDL) cholesterol, but not necessarily elevated low-density lipoprotein (LDL) cholesterol (2); and may have high, or even very high, cardiovascular disease (CVD) risk (3). Dietary choices may produce profound and rapid effects on plasma lipoproteins, even under isocaloric conditions, and is thus possible to determine with high evidence in well-conducted short-term randomized controlled trials (RCTs). When several foods with cholesterol-lowering effects are combined (e.g., in the Portfolio diet) in strictly controlled settings, effects approaching 30% may be achieved on the group level (4). However, the effects of dietary fat quality (e.g., saturated and polyunsaturated fat) on LDL cholesterol and levels of apolipoprotein B are less pronounced in individuals with obesity than without (5). Considering the strong association between NAFLD and obesity, it can thus be speculated that the effect of dietary fat type may be less effective in individuals with compared to without NAFLD (due to concomitant overweight), however this requires further investigation. Factors proposed to blunt the effectiveness of dietary fat modification on LDL cholesterol in obesity (and thus speculatively in NAFLD) are increased inflammation, insulin resistance and endogenous cholesterol synthesis (6). Triglycerides may also be causally associated with CVD risk and can be clearly affected by diet and/or weight loss, although by distinctly different food choices than regarding hypercholesterolemia (7). The mechanisms through which foods influence plasma lipoproteins are partly unknown and may also be independent of the overall dietary fatty acid composition (8). The main routes involve intestinal absorption and excretion of lipids (including bile acids), or hepatic synthesis or clearance from the circulation. However, it is unknown whether hepatic steatosis modifies these effects. For instance, foods affecting gut microbiota (e.g., probiotics and prebiotics) may be of special importance. Also, foods affecting intestinal cholesterol uptake (e.g., foods enriched with phytosterols and/or stanols) could theoretically have less importance in NAFLD compared with foods believed to mainly exert direct effects on the liver, e.g., foods high in polyunsaturated fatty acids (PUFA). Thus, we aimed to systematically evaluate the literature for the effects of foods on blood lipids in persons with NAFLD.

## Methods

A systematic search for systematic reviews and RCTs was performed, in PubMed, Cochrane Database of Systematic Reviews, and Cochrane Central Register of Controlled Trials, from inception through March 2020. The PICO and full search strategy are provided in Table 1. Included search terms were selected based on dyslipidemia guidelines and studies in other populations. The target population was adults with NAFLD, not taking lipid-lowering medication. In order to be included, studies must include information on NAFLD diagnosis or provide other data of liver fat content of participants, and a clear majority of participants must conform with inclusion criteria. Interventions of interest were foods and dietary patterns, but not single nutrients (not clearly associated with specific foods), supplements, nor weight loss diets. As comparison, other foods or background diets were acceptable, but not different doses of the same food. The outcomes of interest were LDL cholesterol, total cholesterol, HDL cholesterol, apolipoprotein B, apolipoprotein AI, triglycerides, non-HDL cholesterol, oxidized LDL cholesterol, and other lipoproteins and subfractions. If data was not reported, authors were not contacted for additional information, however, for included studies, if data was ambiguous, authors were contacted for clarification. Bibliographies of included RCTs were screened for additional studies. Language was restricted to English. The study protocol is available at www.crd.york.ac.uk/PROSPERO, identifier CRD42018089661. No ethics approval was required.

**Table 1:** PICO and search strategy.

**Population:** Adults with NAFLD, not taking lipid-lowering medication
**Intervention:** Intake of foods and dietary patterns, but not weight loss diets, supplements, nor individual nutrients.
**Control:** Background diet or comparison foods, but not lower dose of the same food.
**Outcomes:** Total cholesterol, LDL cholesterol, HDL cholesterol, triglycerides, apo B, apo AI, non-HDL cholesterol, other lipoproteins and subfractions, oxidized LDL cholesterol.
**Searches in PubMed, Cochrane database of systematic reviews and cochrane central register of controlled trials, through March 2020**
Filter: RCTs, systematic reviews and meta-analyses. Modified for Pubmed by: systematic review[tiab] OR systematic literature review[tiab] OR systematic scoping review[tiab] OR systematic narrative review[tiab] OR systematic qualitative review[tiab] OR systematic evidence review[tiab] OR systematic quantitative review[tiab] OR systematic meta-review[tiab] OR systematic critical review[tiab] OR systematic mixed studies review[tiab] OR systematic mapping review[tiab] OR systematic cochrane review[tiab] OR systematic search and review[tiab] OR systematic integrative review[tiab] OR systematic review[pt] OR randomized controlled trial[pt] OR randomized[tiab] OR randomized[tiab] AND controlled[tiab] AND trial[tiab] OR meta-analy*[tiab] OR metaanaly*[tiab] OR Meta-Analysis[pt] Title/Abstract: Lipidemia* OR Dyslipidemia* OR Hyperlipidemia* OR Cholesterol* OR Lipoprotein* OR MeSH Terms: Dyslipidemias OR Hyperlipidemias OR Cholesterol OR “Cholesterol, LDL” OR Lipoproteins AND Title/Abstract: Food OR Foods OR Diet OR Diets OR Dietary OR Coffee OR Butter OR Oil OR Saturated fat* OR Unsaturated fat* OR Monounsaturated fat* OR Polyunsaturated fat* OR Fish OR Soluble fiber* OR Viscous fiber* OR soluble fiber* OR viscous fiber* OR Oats OR Barley OR Psyllium OR Flaxseeds OR Soy OR Almonds OR Nuts OR Plant sterol* OR Plant stanol* OR Phytosterol* OR Phytosteroid* OR Tomato OR Sugar OR Carbohydrate* OR Alcohol OR Omega-3 OR MeSH terms: Food OR Diet OR Coffee OR Butter OR “Plant Oils” OR “Fatty Acids” OR “Fatty Acids, Unsaturated” OR “Fatty Acids, Monounsaturated” OR“ Fatty Acids, Omega-3” OR “Fish Products” OR “Fish Oils” OR Avena OR Hordeum OR “Edible Grain” OR Psyllium OR Flax OR “Soy Foods” OR “Prunus dulcis” OR Nuts OR Phytosterols OR “Lycopersiconesculentum” OR Sugars OR Carbohydrates OR Alcohols AND Title/Abstract: NAFLD OR “Fatty liver” OR “Liver steatosis” OR MeSH terms: “Non-alcoholic Fatty Liver Disease”

apo, apolipoprotein; HDL, high-density lipoprotein; LDL, low-density lipoprotein; NAFLD, non-alcoholic fatty liver disease; MUFA, monounsaturated fatty acids; RCT, randomized controlled trial. *Wildcard caracter.

## Study Selection, Data Extraction, Risk of Bias, and Evidence Assessments

Two reviewers screened abstracts and in cases of disagreement, the paper was included for full-text review. From RCTs that fulfilled inclusion criteria, data was extracted by at least two researchers using standardized forms including: author, year, study type (parallel/crossover), population (NAFLD/other), *n* analyzed overall and per group, % male, age in years (mean ± SD and/or range), intervention/daily dose (mean ± SD and/or range), control/daily dose (mean ± SD and/or range), effects on lipids (mean difference, 95%CI and/or end mean for intervention and control groups), duration, country, and funding. Risk of bias in RCTs was assessed by two authors using the SBU Risk of Bias tool for intervention studies (version 6 May 2020). The quality of evidence was evaluated using The Grading of Recommendations Assessment, Development, and Evaluation (GRADE), by pre-determined criteria for risk of bias, inconsistency, indirectness, imprecision, publication bias, large effects, dose response relationships, and opposing bias. For instance, evidence was downgraded (-1) for inconsistency if results were not replicated, as consistent results of low heterogeneity are impossible to attain from a single RCT. In indeterminate cases, discussions were extended among all authors and overall judgments were employed.

## Data Synthesis and Analysis

Results from included RCTs were included in meta-analyses, when considered appropriate, using ReviewManager 5.3 software. The Cochrane Handbook (9) was adhered to. In cases of at least moderate (I^2^>50%) unexplained heterogeneity, random effects models were preferred. If mean changes from baseline and their standard deviations were unavailable and impossible to calculate, end-of-study means and standard deviations were used. Cross-over studies were included together with parallel studies, with modified weights, calculated from available data. In cases where the variances of mean differences were unavailable, a conservative correlation *R* = 0.5 was imputed and standard deviations adjusted accordingly. Mg/dL was converted to mmol/L by multiplication with 0.02586 for cholesterol and 0.01129 for triglycerides. Data is presented as mean±SD. *P*-values > 0.05 were considered as non-significant (NS).

## Results

**Figure 1:**
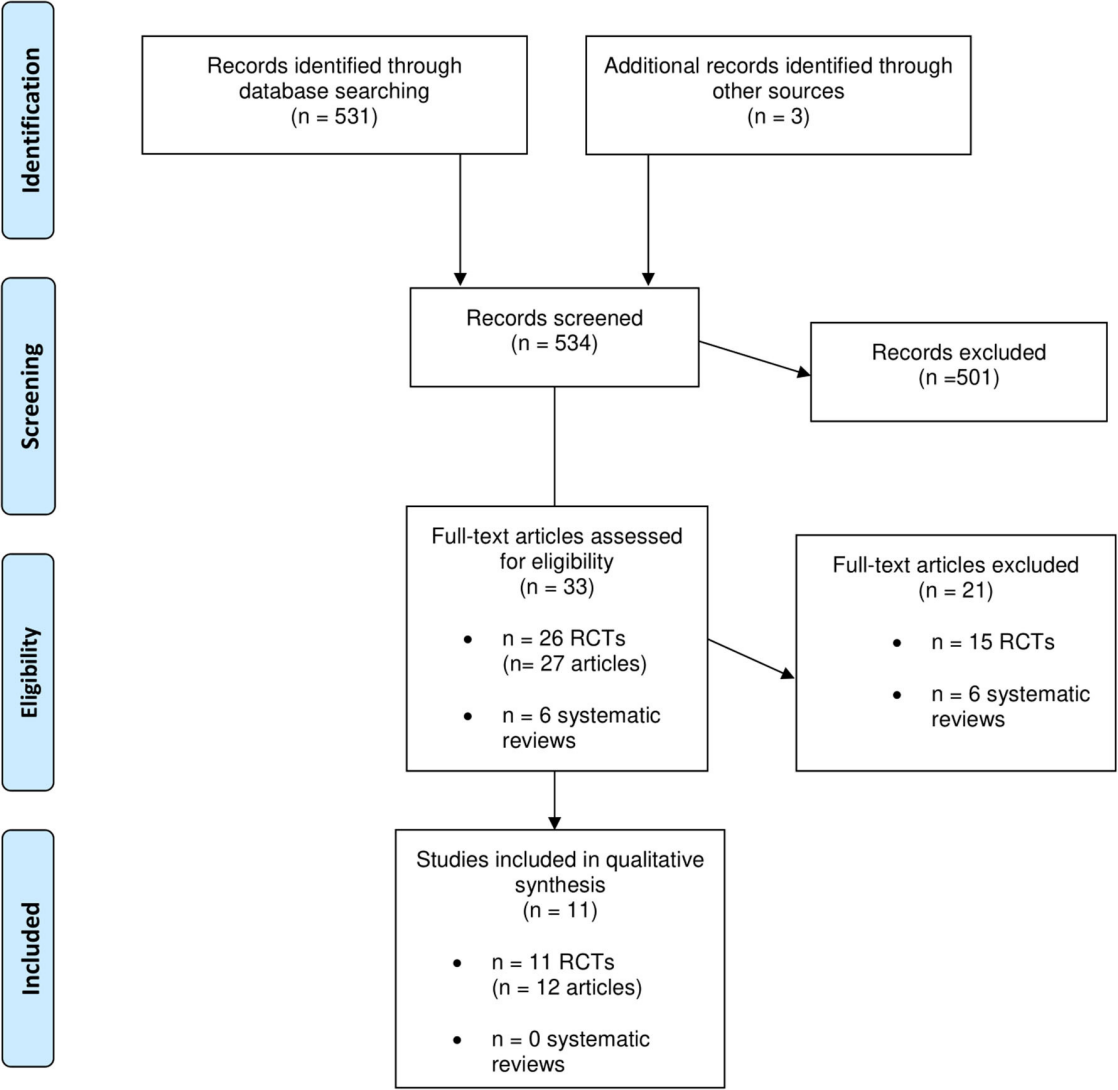
Flow diagram of the studies retrieved for the review.

**Figure 2:**
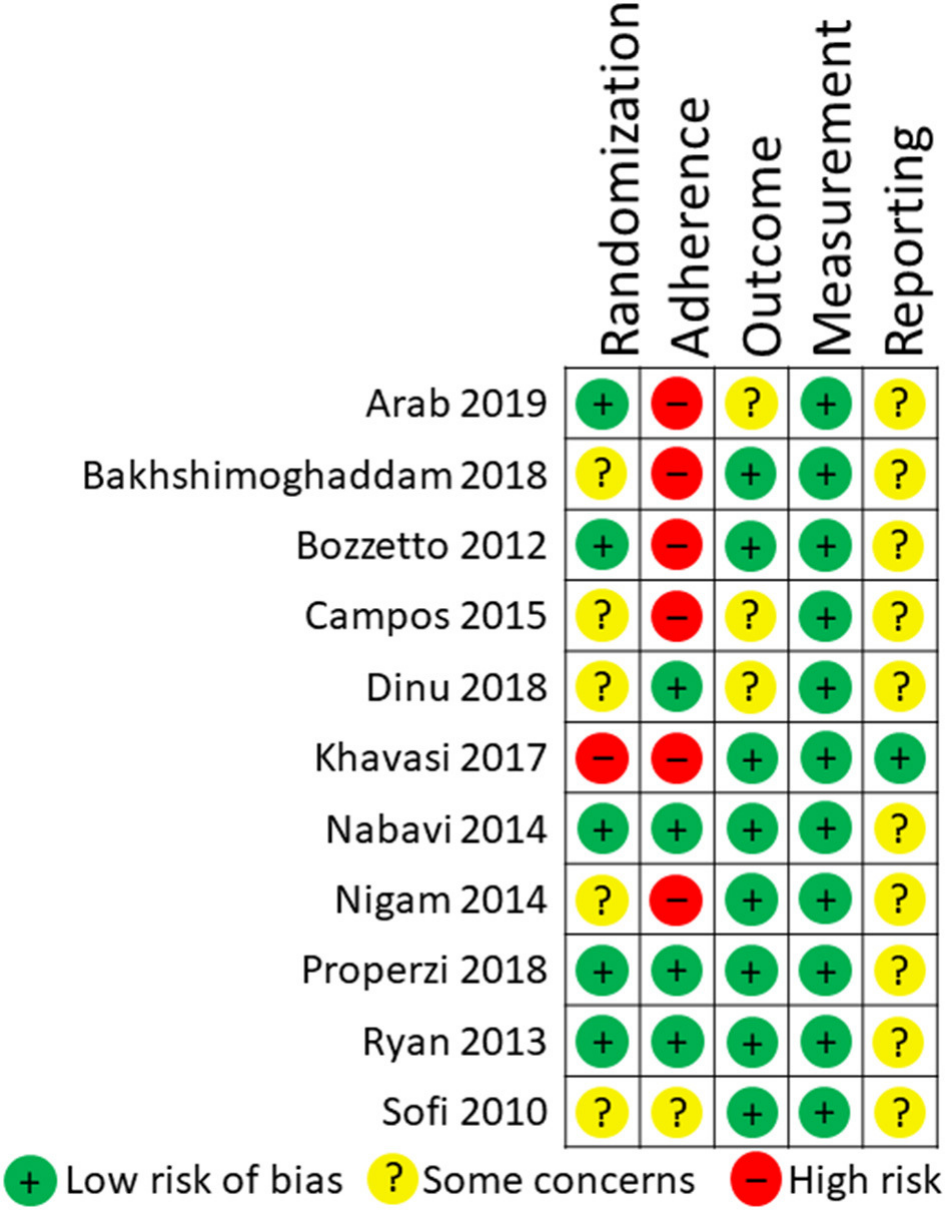
Risk of bias in the 11 included RCTs.

**Figure 3:**
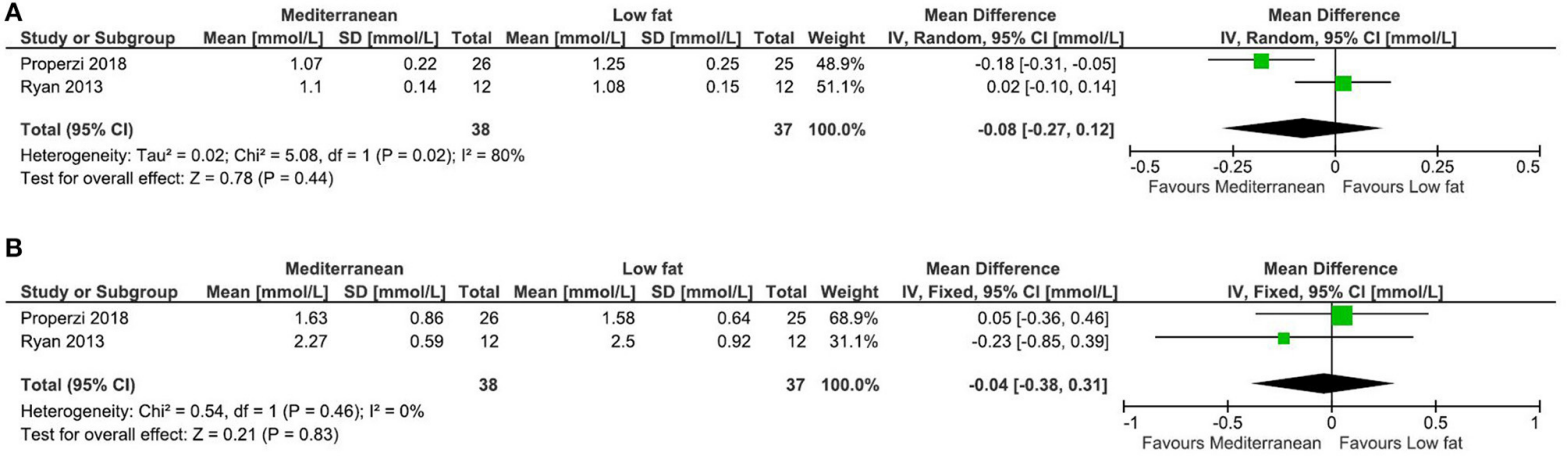
Meta-analysis of Mediterranean vs. Low fat dietary pattern for blood lipids in NAFLD. **(A)**. Forest plot for the effects on HDL Cholesterol. **(B)**. Forest plot for the effects on Triglycerides. Ryan et al. (20) is a cross-over study and its variance has been adjusted accordingly, in order to increase its weighting.

Six systematic reviews and 26 RCTs were evaluated in full text. No systematic reviews and 11 RCTs (12 publications) fulfilled inclusion criteria (Figure 1). The characteristics of the 11 included RCTs are presented in Table 2. The excluded papers are presented, with reasons, in Table 3. The overall risk of bias was considered as Low in three RCTs, Some concerns in six RCTs, and High in two RCTs (Table 2 and Figure 2). Four RCTs compared dietary patterns and the remaining seven compared food items. The overall results on blood lipids and the evidence gradings for each available outcome are presented in Table 4. Two studies were considered appropriate to include in meta-analyses, as they both compared a Mediterranean with a Low-fat dietary pattern (Figure 3).

**Table 2:** Characteristics of included 11 RCTs on the effects of foods on blood lipids in NAFLD.

**References**	**Design**	***n* analyzed (per group), % males**	**Intervention, daily dose**	**Comparison, daily dose**	**Duration**	**Country**	**Funding**	**Risk of bias**
Arab et al. (10)	Parallel	*n* = 69 (36 + 33), 32% males	Healthy dietary pattern (fruit, vegetables, complex carbohydrates, low fat dairy, healthy fats, white meat, and fish; avoid unhealthy fats and refined carbohydrates)	Usual care (reduce calories, carbohydrates, and fat)	2 months	Iran	Isfahan University	Some concerns
Bakhshimoghaddam et al. (11)	Parallel	*n* = 102 (34 + 34 + 34), 49% males	300 g synbiotic yogurt (*B. animalis* and inulin) + advice on healthy lifestyle	300 g conventional yogurt (*Streptococcus thermophilus* and *Lactobacillus delbrueckii* subsp. Bulgaricus) + advice on healthy lifestyle; or advice on healthy lifestyle only	24 weeks	Iran	Urmia University	Some concerns
Bozzetto et al. (12, 13)	Parallel	*n* = 17 (8 + 9), 76% males	MUFA diet with olive oil, fat 42E% (MUFA 27E%, SFA 6.7E%, PUFA 4.6E%), CHO 40E%	CHO/fiber/Low-GI diet: fat 28E% (MUFA 16E%, SFA 6E%, PUFA 3.8E%), CHO 53E%	8 weeks	Italy	EU funding, Italian diabetes society	Some concerns
Campos et al. (14)	Parallel	*n* = 15 (8 + 7), 52% males	Artificially sweetened beverages, at least 660 ml (replacing habitual consumption of sugar-sweetened beverages)	Sugar-sweetened beverages, continue with habitual intake of at least 660 ml	12 weeks	Switzerland	Swiss national foundation for science, Fondation Raymond Berger pour la recherche sur le diabete et les maladies metaboliques	High risk
Dinu et al. (15)	Parallel	*n* = 40 (20 + 20), 30% males	Kamut khorasan wheat, 71 g pasta, 21 g bread, 36 g crackers, 36 g biscuits	Modern wheat, 71 g pasta, 21 g bread, 36 g crackers, 36 g biscuits	3 months	Italy	Kamut enterprises of Europé	Some concerns
Khavasi et al. (16)	Parallel	*n* = 44 (22 + 22), 34% males	Pickled caper fruit, 40–50 gram + lifestyle changes	Lifestyle changes only	12 weeks	Iran	Tabriz University	High risk
Nabavi et al. (17)	Parallel	*n* = 72 (36 + 36), 49% males	Probiotic yogurt (*L. acidophilus* La5 and *B. lactis* Bb12 + *Lactobacillus bulgaricus* and *Streptococcus thermophilus*), 300 g	Conventional yogurt (*L. bulgaricus* and *Streptococcus thermophilus*), 300 g	8 weeks	Iran	Tabriz University	Low risk
Nigam et al. (18)	Parallel	*n* = 93 (30 + 33 + 30), 100% males	Olive oil, 20 g	Canola oil, 20 g or commonly used oil (soybean or safflower), 20 g	6 months	India	Dalmia Continental Pvt. Ltd.	Some concerns
Properzi et al. (19)	Parallel	*n* = 48 (24 + 24), 52% males	Mediterranean diet + 27 g nuts (almonds or walnuts) and 27 mL olive oil	Low-fat diet + 36 g natural muesli and 7 g low-fat snack bar	12 weeks	Australia	Australian government research training program scholarship	Low risk
Ryan et al. (20)	Crossover	*n* = 12, 50% males	Cretan Mediterranean diet 40/40/20%E fat/CHO/protein (*ad libitum*, food supplied)	Low fat-high carbohydrate diet 30/50/20%E fat/CHO/protein (*ad libitum*, food supplied)	6 weeks	Australia	NHMRC Neil Hamilton Fairley Fellowship/University of Melbourne	Low risk
Sofi et al. (15)	Parallel	*n* = 11 (6 + 5), 82% males	Olive oil enriched with n-3 PUFA, 6.5 ml (0.83 g n-3 PUFA, of which 0.47 g EPA and 0.24 g DHA, supplied)	Olive oil, 6.5 ml (supplied)	12 months	Italy	N/A	Some concerns

*CHO, carbohydrate; DHA, docosahexaenoic acid; EPA, eicosapentaenoic acid; GI, glycemic index; HDL, high-density lipoprotein; LDL, low-density lipoprotein; NAFLD, non-alcoholic fatty liver disease; MUFA, monounsaturated fatty acids; PUFA, polyunsaturated fatty acids; RCT, randomized controlled trial; SFA, saturated fatty acids*.

**Table 3:** Excluded six systematic reviews and 15 RCTs, with reasons.

**Study type**	**Food/comparison**	**References**	**Reason for exclusion**
Systematic review	Fructose	Sievenpiper et al. (21)	Nutrient, not food-specific
Systematic review	Probiotics	Lirussi et al. (22)	No RCTs identified
Systematic review	Probiotics	Liu et al. (23)	Supplements, not foods
Systematic review	Probiotics	Ma et al. (24)	Supplements, not foods
Systematic review	Probiotics	Xiao et al. (25)	Supplements, not foods
Systematic review	Probiotics, prebiotics, or synbiotics	Loman et al. (26)	Supplements, not foods
RCT	Alternate-day fasting vs. time-restricted feeding	Cai et al. (27)	Eating patterns, not foods or dietary patterns
RCT	*Bifidobacterium longum* with fructo-oligosaccharides	Malaguarnera et al. (28)	Supplement, not food
RCT	Catechin-enriched green and oolong tea	Venkatakrishnan et al. (29)	Wrong study population (not NAFLD)
RCT	Combined nutraceutical containing berberine, chlorogenic acid, and tocotrienols	Cicero et al. (30)	Supplement, not food
RCT	Fructose	Jin et al. (31)	Wrong study population (not adults)
RCT	Low-GI Mediterranean diet	Misciagna et al. (32)	Data not provided on blood lipids
RCT	Low-carbohydrate vs. low-fat diet	Jang et al. (33)	Data not provided on food intake; and intentional weight loss
RCT	Low-carbohydrate vs. low-fat diet	Rodríguez-Hernández et al. (34)	Aiming for weight loss
RCT	Low-fat/low-saturated fat/low-GI diet vs. high-fat/high-saturated fat/high-GI diet	Utzschneider et al. (35)	Wrong study population (not NAFLD)
RCT	Mediterranean diet vs. Mediterranean lifestyle	Katsagoni et al. (36)	Aiming for weight loss
RCT	Milk- and soya-phospholipids	Weiland et al. (37)	Supplements, not foods; and no information on NAFLD status
RCT	Northern berries	Lehtonen et al. (38)	No information on NAFLD status
RCT	Probiotic yogurt	Nabavi et al. (39)	Not written in English
RCT	Sugar	Umpleby et al. (40)	Different doses only, no comparison food
RCT	Whole-grain wheat vs. refined wheat	Schutte et al. (41)	Wrong study population (not NAFLD)

*GI, glycemic index; NAFLD, non-alcoholic fatty liver disease; RCT, randomized controlled trial*.

**Table 4:** GRADE table of the effects of dietary patterns and foods on blood lipids in NAFLD.

**Food/comparison**	**Type and no. of studies**	**Outcome**	**Effect**	**GRADE evidence**
Healthy dietary pattern vs. usual care	1 RCT (10)	Total cholesterol	Reduction	Very low (⊕)^a^^,^^b^^,^^d^
		LDL cholesterol	Reduction	Very low (⊕)^a^^,^^b^^,^^d^
		HDL cholesterol	No clear effect	Very low (⊕)^a^^,^^b^^,^^d^
		Triglycerides	Reduction	Moderate (⊕⊕⊕)^a^^,^^b^^,^^e^
Mediterranean diet vs. Low-fat diet	1 RCT (19)	Total cholesterol	Reduction	Low (⊕⊕)^b^^,^^d^
		LDL cholesterol	No clear effect	Low (⊕⊕)^b^^,^^d^
	2 RCTs (19, 20)	HDL cholesterol	No clear effect	Low (⊕⊕)^b^^,^^d^
		Triglycerides	No clear effect	Low (⊕⊕)^d−2^
MUFA diet vs. High CHO/High-fiber/Low-GI diet	1 RCT (12, 13)	TC	Reduction	Very low (⊕)^a^^,^^b^^,^^d−2^
		LDLC	Reduction	Very low (⊕)^a^^,^^b^^,^^d−2^
		HDLC	No clear effect	Very low (⊕)^a^^,^^b^^,^^d−2^
		TG	Reduction	Very low (⊕)^a^^,^^b^^,^^d−2^
Artifically sweetened beverages vs. sugar-sweetened beverages	1 RCT (14)	Total cholesterol	No clear effect	Very low (⊕)^a−2^^,^^b^^,^^d−2^
		HDL cholesterol	No clear effect	Very low (⊕)^a−2^^,^^b^^,^^d−2^
		Triglycerides	Increase	Very low (⊕)^a−2^^,^^b^^,^^d−2^
Kamut khorasan vs. modern wheat	1 RCT (42)	Total cholesterol	Reduction	Very low (⊕)^a^^,^^b^^,^^d^
		LDL cholesterol	Reduction	Very low (⊕)^a^^,^^b^^,^^d^
		HDL cholesterol	No clear effect	Very low (⊕)^a^^,^^b^^,^^d^
		Triglycerides	Reduction	Very low (⊕)^a^^,^^b^^,^^d^
Olive oil vs. canola oil	1 RCT (18)	HDL cholesterol	No clear effect	Very low (⊕)^a^^,^^b^^,^^d^
		Triglycerides	Increase	Very low (⊕)^a^^,^^b^^,^^d^
Olive oil vs. commonly used oil (soybean/safflower)	1 RCT (18)	HDL cholesterol	Increase	Very low (⊕)^a^^,^^b^^,^^c^^,^^d^
		Triglycerides	No clear effect	Very low (⊕)^a^^,^^b^^,^^d^
Canola oil vs. commonly used oil (soybean/safflower)	1 RCT (18)	HDL cholesterol	Increase	Very low (⊕)^a^^,^^b^^,^^c^^,^^d^
		Triglycerides	Reduction	Very low (⊕)^a^^,^^b^^,^^d^
Olive oil enriched with n-3 PUFA vs. olive oil	1 RCT (15)	Total cholesterol	No clear effect	Very low (⊕)^a^^,^^b^^,^^c^^,^^d−2^
		LDL cholesterol	No clear effect	Very low (⊕)^a^^,^^b^^,^^c^^,^^d−2^
		HDL cholesterol	Increase	Very low (⊕)^a^^,^^b^^,^^c^^,^^d−2^
		Triglycerides	Reduction	Very low (⊕)^a^^,^^b^^,^^c^^,^^d−2^^,^^e^
Pickled caper fruit vs. control (background) diet	1 RCT (16)	Total cholesterol	No clear effect	Very low (⊕)^a(−2)^^,^^b^^,^^d^
		LDL cholesterol	No clear effect	Very low (⊕)^a(−2)^^,^^b^^,^^d^
		HDL cholesterol	No clear effect	Very low (⊕)^a(−2)^^,^^b^^,^^d^
		Triglycerides	No clear effect	Very low (⊕)^a(−2)^^,^^b^^,^^d^
Probiotic yogurt (enriched with *L. acidophilus* La5 and *B. lactis* Bb12) vs. conventional yogurt (*L. bulgaricus* and *Streptococcus thermophilus*)	1 RCT (17)	Total cholesterol	Reduction	Moderate (⊕⊕⊕)^b^^,^^d^^,^^e^
		LDL cholesterol	Reduction	Low (⊕⊕)^b^^,^^d^
		HDL cholesterol	No clear effect	Low (⊕⊕)^b^^,^^d^
		Triglycerides	Reduction	Low (⊕⊕)^b^^,^^d^
Synbiotic yogurt (*B. animalis* and inulin) vs. conventional yogurt	1 RCT (11)	Total cholesterol	Reduction	Low (⊕⊕)^a^^,^^b^^,^^d^^,^^e^
		LDL cholesterol	Reduction	Low (⊕⊕)^a^^,^^b^^,^^d^^,^^e^
		HDL cholesterol	No clear effect	Very low (⊕)^a^^,^^b^^,^^d^
		Triglycerides	Reduction	Low (⊕⊕)^a^^,^^b^^,^^d^^,^^e^
Synbiotic yogurt (*B. animalis* and inulin) vs. control	1 RCT (11)	Total cholesterol	Reduction	Low (⊕⊕)^a^^,^^b^^,^^d^^,^^e^
		LDL cholesterol	Reduction	Low (⊕⊕)^a^^,^^b^^,^^d^^,^^e^
		HDL cholesterol	No clear effect	Very low (⊕)^a^^,^^b^^,^^d^
		Triglycerides	Reduction	Low (⊕⊕)^a^^,^^b^^,^^d^^,^^e^
Conventional yogurt (*Streptococcus thermophilus* and *L. delbrueckii* subsp. Bulgaricus) vs. control	1 RCT (11)	Total cholesterol	Reduction	Very low (⊕)^a^^,^^b^^,^^d^
		LDL cholesterol	Reduction	Very low (⊕)^a^^,^^b^^,^^d^
		HDL cholesterol	No clear effect	Very low (⊕)^a^^,^^b^^,^^d^
		Triglycerides	No clear effect	Very low (⊕)^a^^,^^b^^,^^d^

*CHO, carbohydrate; GI, glycemic index; HDL, high-density lipoprotein; LDL, low-density lipoprotein; NAFLD, non-alcoholic fatty liver disease; MUFA, monounsaturated fatty acids; RCT, randomized controlled trial*.

Downgraded for

a *Risk of bias*,

b *Inconsistency*,

c *Indirectness*,

d Imprecision, or upgraded for

e *Large effects*.

## Dietary Patterns

### Healthy Dietary Pattern vs. Usual Care

In the RCT by Arab et al. (10), there were significant reductions in both total cholesterol (from 5.56 ± 1.6 to 5.01 ± 1.8 mmol/l in intervention vs. from 4.81 ± 0.92 to 4.91 ± 0.89 mmol/l in control group, *P* = 0.04) and triglycerides (from 2.46 ± 1.9 to 1.86 ± 0.98 vs. from 2.11 ± 1.7 to 2.21 ± 1.4 mmol/L, *P* = 0.002). There was a tendency toward reduced LDL cholesterol (from 3.11 ± 1.0 to 2.83 ± 1.1 vs. from 2.87 ± 0.84 to 2.96 ± 0.79 mmol/L, *P* = 0.46), but no clear effect for HDL cholesterol (from 1.20 ± 0.29 to 1.16 ± 0.20 vs. from 1.12 ± 0.26 to 1.07 ± 0.20 mmol/L, *P* = 0.12). The quality of evidence for the effect on triglycerides was considered as Moderate, upgraded for large effects, whereas all other comparisons were further downgraded to Very low evidence, due to high imprecision.

### Mediterranean Diet vs. Low-Fat Diet

From the RCTs by Properzi et al. (19) and Ryan et al. (20), effects on HDL cholesterol and triglycerides were combined in meta-analyses, but there were no clear effects (Figure 3). Effects on total and LDL cholesterol were only reported in the study by Properzi et al. (19), with an indicated slight reduction for total cholesterol (from 4.78 ± 1.3 to 4.53 ± 1.3 vs. from 5.23 ± 0.90 to 5.15 ± 1.1 mmol/L, NS), but no meaningful effect on LDL cholesterol (from 2.83 ± 1.2 to 2.73 ± 1.1 vs. from 3.20 ± 0.78 to 3.17 ± 0.86 mmol/L, NS). The evidence was considered as Low for all outcomes.

### High-Monounsaturated Fatty Acid Diet vs. High-Carbohydrate, High-Fiber, Low-Glycemic Index Diet

In the RCT by Bozzetto et al. (12, 13), there were tendencies (all NS) toward reductions after the high-monounsaturated fatty acid (MUFA) diet vs. high-carbohydrate (high-CHO) diet, for total (from 4.32 ± 0.65 to 4.29 ± 0.59 vs. from 4.06 ± 0.98 to 4.24 ± 1.2 mmol/L) and LDL cholesterol (from 2.85 ± 0.52 to 2.82 ± 0.54 vs. from 2.53 ± 0.75 to 2.77 ± 1.0 mmol/L), and for triglycerides (from 1.38 ± 0.42 to 1.29 ± 0.37 vs. from 1.24 ± 0.77 to 1.48 ± 1.2 mmol/L). There was no clear effect on HDL cholesterol (from 0.91 ± 0.2 to 0.93 ± 0.1 vs. from 0.96 ± 0.2 to 0.96 ± 0.2 mmol/L). The evidence was considered as Very low. In the report from 2014 (10), results were reported on postprandial lipids combined with 19 (8 + 11 per group) additional subjects that were also prescribed exercise training. Post-prandial (after 2, 4, and 6 h, presented as figure) triglycerides and cholesterol in plasma and chylomicrons/very-low-density lipoproteins were increased after the MUFA diet (P<0.05) and reduced after the high-CHO diet (*P* < 0.05). We did not perform evidence gradings for these outcomes, as they were not clearly pre-specified.

## Foods

### Artificially vs. Sugar-Sweetened Beverages

In the RCT by Campos et al. (14), triglycerides tended to increase by artificially (from 1.35 ± 0.2 to 1.5 ± 0.3 mmol/L) vs. sugar-sweetened beverages (from 2.0 ± 0.6 to 1.3 ± 0.2 mmol/L), however based on only 8+7 individuals with steatosis, and the evidence was graded as Very low. There were no clear effects on total (from 4.3 ± 0.3 to 4.2 ± 0.4 vs. from 4.3 ± 0.3 to 4.2 ± 0.2 mmol/L) or HDL cholesterol (from 1.15 ± 0.08 to 1.10 ± 0.07 vs. from 1.11 ± 0.07 to 1.14 ± 0.08 mmol/L), also with Very low evidence.

### Kamut Khorasan vs. Modern Wheat

In the RCT by Dinu et al. (42), there were tendencies toward reductions in total (from 5.89 ± 1.0 to 5.39 ± 0.94 vs. from 5.51 ± 0.65 to 5.56 ± 0.73 mmol/L, *P* = 0.096) and LDL cholesterol (from 3.76 ± 0.94 to 3.46 ± 0.85 vs. from 3.36 ± 1.1 to 3.37 ± 0.92 mmol/L, *P* = 0.12), and in triglycerides (from 1.57 ± 0.97 to 1.36 ± 0.51 vs. from 1.41 ± 0.56 to 1.50 ± 0.57 mmol/L *P* = 0.16), in the kamut vs. modern wheat group. There was no clear effect on HDL cholesterol (from 1.43 ± 0.33 to 1.39 ± 0.35 vs. from 1.45 ± 0.44 to 1.37 ± 0.35 mmol/L, *P* = 0.99). The evidence was considered as Very low for all outcomes.

### Olive Oil vs. Canola Oil vs. Commonly Used Oil (Soybean or Safflower)

The RCT by Nigam et al. (18) had three parallel arms, which besides dietary oils received similar lifestyle counseling including exercise. For HDL cholesterol, there were tendencies toward increases for olive (from 0.98 ± 0.11 to 1.07 ± 0.12 mmol/L) and canola (from 1.02 ± 0.14 to 1.05 ± 0.15 mmol/L), vs. commonly used oil (from 1.02 ± 0.13 to 0.91 ± 0.14 mmol/L), but no meaningful difference for olive vs. canola oil. For triglycerides, there were tendencies toward an increase for olive (from 2.04 ± 0.93 to 1.92 ± 0.14 mmol/L) vs. canola oil (from 2.11 ± 0.93 to 1.75 ± 0.55 mmol/L), toward no clear effect for olive vs. commonly used oil (from 2.08 ± 1.1 to 2.06 ± 1.2 mmol/L), and toward a reduction for canola vs. commonly used oil. The evidence was considered as Very low for all comparisons and outcomes.

### Olive Oil Enriched With n-3 PUFA vs. Olive Oil

In the RCT by Sofi et al. (15), there was an increase in HDL cholesterol (from 1.15 ± 0.14 to 1.56 ± 0.24 vs. from 1.13 ± 0.12 to 1.07 ± 0.05 mmol/L, *P* = 0.03) and a reduction in triglycerides (from 1.86 ± 0.97 to 1.50 ± 0.72 vs. from 1.61 ± 0.28 to 1.84 ± 0.34 mmol/L, *P* = 0.04) in the enriched vs. regular olive oil group. No clear differences were seen for total (from 5.53 ± 0.72 to 5.51 ± 0.60 vs. from 5.11 ± 0.57 to 4.99 ± 0.58 mmol/L, *P* = 0.9) or LDL cholesterol (from 3.40 ± 0.64 to 3.45 ± 0.84 vs. from 3.24 ± 0.50 to 3.08 ± 0.60 mmol/L, *P* = 0.2). The evidence was considered as Very low for all outcomes.

### Pickled Caper Fruit vs. No Food

In the RCT by Khavasi et al. (16), there were no clear effects on total (from 4.95 ± 1.2 to 4.75 ± 1.2 vs. from 4.31 ± 0.96 to 3.94 ± 0.93 mmol/L, *P* = 0.09), LDL (from 2.90 ± 0.93 to 2.67 ± 0.73 vs. from 2.00 ± 1.2 to 1.68 ± 1.1 mmol/L, *P* = 0.03), or HDL cholesterol (from 0.99 ± 0.21 to 1.01 ± 0.22 vs. from 1.14 ± 0.13 to 1.14 ± 0.16 mmol/L, *P* = 0.3), or on triglycerides (from 2.14 ± 0.63 to 2.15 ± 0.90 vs. from 2.54 ± 1.0 to 2.44 ± 1.1 mmol/L, *P* = 0.053). Some data on variances were estimated because of implausible data in table. The evidence was considered as Very low for all outcomes.

### Probiotic (*L. acidophilus* La5 and *B. lactis* Bb12) vs. Conventional Yogurt

In the RCT by Nabavi et al. (17), there was a reduction in total (from 5.08 ± 1.0 to 4.46 ± 1.1 vs. from 5.14 ± 0.82 to 5.25 ± 0.87 mmol/L) and LDL cholesterol (from 3.11 ± 0.91 to 2.58 ± 0.56 vs. from 2.88 ± 0.77 to 2.85 ± 0.70 mmol/L), and at least a tendency for reduced triglycerides (from 2.19 ± 0.72 to 1.95 ± 0.77 vs. from 2.23 ± 0.87 to 2.33 ± 0.90 mmol/L) by the probiotic vs. conventional yogurt (*P*-values N/A for mean differences in change). No clear effect was observed for HDL cholesterol (from 1.23 ± 0.28 to 1.27 ± 0.32 vs. from 1.24 ± 0.25 to 1.31 ± 0.25 mmol/L). The evidence was Moderate for total cholesterol (upgraded for large effects) and Low for all other outcomes.

### Synbiotic (*B. animalis* and Inulin) vs. Conventional Yogurt vs. No Food

In the RCT by Bakhshimoghaddam et al. (11), there were reductions in total and LDL cholesterol, and triglycerides by the synbiotic compared with conventional yogurt or control group (data presented as graphs), with Low evidence. For HDL cholesterol, there was no clear effect for any comparison, with Very low evidence. For the conventional yogurt compared with control group, there were reductions in total and LDL cholesterol, but not for HDL cholesterol or triglycerides, with Very low evidence for all outcomes.

## Discussion

In this systematic review of the effects of foods on blood lipids in individuals with NAFLD, no systematic reviews and only 11 RCTs fulfilled the inclusion criteria. No foods or dietary patterns modifies blood lipids with High quality evidence in NAFLD. With Moderate evidence, a Healthy dietary pattern reduces fasting triglycerides, compared with standard care. Also with Moderate evidence, a probiotic yogurt, enriched with *Lactobacillus acidophilus* La5 and *Bifidobacterium lactis* Bb12, decreases total cholesterol, compared with conventional yogurt, and with Low evidence, reduces LDL cholesterol and triglycerides. Also with Low evidence, a synbiotic yogurt containing *Bifidobacterium animalis* and inulin reduces total and LDL cholesterol, and triglycerides. Finally, with Low evidence, a Mediterranean dietary pattern (high in unsaturated fatty acids) reduces total cholesterol (but not other lipoproteins) compared with a Low-fat dietary pattern. For all other comparisons, the evidence quality was considered as Very low for all effects, or lack thereof.

The limited number of studies discovered was somewhat surprising, given the prevalence and clinical significance of NAFLD. One plausible reason may be that, in most studies on diet and CVD risk factors, liver fat content is not easily attainable and thus overlooked. Also, many patients with NAFLD go undiagnosed, as it usually presents with little symptoms. The effect of dietary fat type (e.g., saturated/polyunsaturated fat) on plasma lipoproteins is diminished in individuals with obesity compared with normal body weight (5). Considering the strong association between NAFLD and obesity, it can thus be speculated that the effect of dietary fat type may be less effective in individuals with compared to without NAFLD (due to concomitant overweight), however this requires further investigation. Factors proposed to blunt the effectiveness of dietary fat modification on LDL cholesterol in obesity (and thus speculatively in NAFLD) are increased inflammation, insulin resistance, and endogenous cholesterol synthesis (6).

An RCT testing the effects of a synbiotic supplement (fructo-oligosaccharides plus *B. animalis*) on liver fat content and liver fibrosis in NAFLD was published during the writing of this manuscript (43). This study showed that this synbiotic combination altered the fecal microbiome but had no effect on liver fat, fibrosis, or plasma lipoproteins compared to prebiotic fructo-oligosaccharides only. This is in contrast to the results reported by Bakhshimoghaddam (11) where a similar synbiotics was administered in the form of yogurt. Whether the mode of administration of synbiotics (supplement vs. yogurt/whole food) modifies the effects on plasma lipoproteins requires further investigation but is not unlikely considering potential matrix effects when part of a whole food.

The present systematic review has limitations. Studies on dietary supplements and individual nutrients were not included in our searches, which could have been relevant e.g.. for determining which bacterial strains (in probiotics) may provide beneficial effects on plasma lipoproteins. Also, we did not search gray literature or contact authors for additional information, and only studies published in English were included. Thus, we cannot rule out that additional relevant studies may exist. In addition, the present study could not confidently determine effect sizes or required daily doses for the included foods, because of the scarce data available.

## Conclusions

The results from the included RCTs are mostly in line with current guidelines for the treatment of dyslipidemia or prevention of CVD (7, 44) in other populations. However, it is not possible from the present data to determine which foods significantly modify blood lipids in NAFLD. The possible beneficial effect of probiotics enriched with certain bacterial strains on plasma lipoproteins warrants further study.

## Data Availability Statement

The original contributions presented in the study are included in the article/supplementary material, further inquiries can be directed to the corresponding author.

## Author Contributions

The study was conceptualized by DI. AR and FR performed screening. AR, FR, and DI performed data extraction. Risk of bias was assessed by FR and DI. Data analysis and grading of the evidence was performed by DI, with support from AR and FR. The paper was written by AR, FR, and DI. All authors contributed to the article and approved the submitted version.

## Conflict of Interest

The authors declare that the research was conducted in the absence of any commercial or financial relationships that could be construed as a potential conflict of interest.
